# Genomic and microbiological analyses of iron acquisition pathways among respiratory and environmental nontuberculous mycobacteria from Hawai’i

**DOI:** 10.3389/fmicb.2023.1268963

**Published:** 2023-11-10

**Authors:** Cara G. Tan, Nicole M. Oberlag, Acelyn E. McGowan, Stephanie N. Dawrs, Yvonne L. Chan, Michael Strong, Nabeeh A. Hasan, Jennifer R. Honda

**Affiliations:** ^1^‘Iolani School, Honolulu, HI, United States; ^2^Center for Genes, Environment and Health, National Jewish Health, Denver, CO, United States; ^3^Rock Canyon High School, Highlands Ranch, CO, United States; ^4^Department of Cellular and Molecular Biology, School of Medicine, The University of Texas Health Science Center at Tyler, Tyler, TX, United States

**Keywords:** nontuberculous mycobacteria, Hawai’i, siderophores, bioinformatics, CAS assay

## Abstract

As environmental opportunistic pathogens, nontuberculous mycobacteria (NTM) can cause severe and difficult to treat pulmonary disease. In the United States, Hawai’i has the highest prevalence of infection. Rapid growing mycobacteria (RGM) such as *Mycobacterium abscessus* and *M. porcinum* and the slow growing mycobacteria (SGM) including *M. intracellulare* subspecies *chimaera* are common environmental NTM species and subspecies in Hawai’i. Although iron acquisition is an essential process of many microorganisms, iron acquisition via siderophores among the NTM is not well-characterized. In this study, we apply genomic and microbiological methodologies to better understand iron acquisition via siderophores for environmental and respiratory isolates of *M. abscessus*, *M. porcinum*, and *M. intracellulare* subspecies *chimaera* from Hawai’i. Siderophore synthesis and transport genes, including mycobactin *(mbt), mmpL/S*, and *esx-3* were compared among 47 reference isolates, 29 respiratory isolates, and 23 environmental Hawai’i isolates. Among all reference isolates examined, respiratory isolates showed significantly more siderophore pertinent genes compared to environmental isolates. Among the Hawai’i isolates, RGM *M. abscessus* and *M. porcinum* had significantly less *esx*-3 and *mbt* genes compared to SGM *M. chimaera* when stratified by growth classification. However, no significant differences were observed between the species when grown on low iron culture agar or siderophore production by the chrome azurol S (CAS) assay *in vitro*. These results indicate the complex mechanisms involved in iron sequestration and siderophore activity among diverse NTM species.

## 1. Introduction

Nontuberculous mycobacteria (NTM) are found in a variety of environmental habitats and biofilms including those collected from showerheads, water faucets, air conditioning units, hospital water faucets, and soil (Honda et al., 2018). There are more than 195 different species of rapid growing mycobacteria (RGM) and slow growing mycobacteria (SGM) that have been identified to date (Parte et al., 2020), yet only a handful of these species are considered opportunistic pathogens that cause pulmonary disease (PD) particularly in susceptible individuals (Falkinham, 1996). NTM PD is becoming a mounting public health concern as the number of cases continue to increase globally (Adjemian et al., 2012).

In the United States (U.S.), Hawai’i had the highest period prevalence of NTM PD calculated at 396 cases/100,000 persons according to a Medicare Part B beneficiary analysis (Adjemian et al., 2012). In our prior research, we demonstrated frequent recovery of viable RGM including *Mycobacterium abscessus* and *Mycobacterium porcinum* from the Hawai’i environment. We also reported the preponderance of the SGM, *Mycobacterium intracellulare* subspecies *chimaera,* among both respiratory and environmental samples from this geographic NTM hot spot (Honda et al., 2016).

Iron is an essential element for microbial growth, used in various biological processes including electron transport, and iron plays a major role in metabolic and cellular pathways including energy generation, DNA replication, transcriptional regulation (Crosa, 1989; Skaar, 2010). In the human body, iron mainly exists in complex forms bound to protein (e.g., hemoprotein) as heme compounds (e.g., hemoglobin or myoglobin), heme enzymes, or nonheme compounds (e.g., flavin-iron enzymes, transferring, and ferritin) (McDowell, 1992; Abbaspour et al., 2014). Minute amounts, i.e., 10^−18^ M concentration of free iron are available or 0.4–0.9 mg Fe/g dry weight in blood or human lungs, respectively (Griffiths et al., 1980; Takemoto et al., 1991). Under this competitive iron-restricted environment, bacteria that have the capacity to sequester and use iron may be selectively advantaged. In contrast, iron is more readily available and in higher quantity in the exogenous environment. Hawai’i soil, for example, contains 95,550 mg/kg of iron on average but iron content varies with rock type as basaltic and mafic rocks range from 56,000–87,000 mg/kg (Hawai’i Department of Health Hazard Evaluation and Emergency Response, 2012).

To acquire exogenous iron, many bacteria exploit siderophores. For mycobacteria, intracellular and extracellular iron-chelating siderophores synergistically scavenge iron from the environment (Gobin et al., 1995) and are critical to pathogenicity (Braun and Winkelmann, 1988; De Voss et al., 2000). Mycobactin (*mbt*) is an intracellular siderophore produced by most mycobacteria, especially pathogenic species, under iron-limiting conditions and is restricted to the cell envelope (De Voss et al., 2000). Based on models for *Mycobacterium tuberculosis (Mtb)*, two gene complexes are needed to synthesize mycobactin: *mbt-1* and *mbt-2. Mbt-1* is comprised of ten genes, ranging from *mbtA* to *mbtJ*. *mbt-2* is comprised of four genes ranging from *mbtK* to *mbtN* (Sritharan, 2016). Exochelin is an extracellular peptide siderophore produced under iron-deficient conditions and exclusive to RGM. The mycobacterial membrane large/small proteins (*mmpL/S*) complex and the type VII secretion system, *esx*-3, are used to scavenge iron from the surroundings, facilitating acquisition of iron from mycobactin (Siegrist et al., 2009; Szekely and Cole, 2016). *esx*-3 involves a homologous gene system relating to protein production for iron acquisition. However, beyond confirmation of their existence, NTM siderophore biology is significantly understudied and most research in this area predates the current genomic era (Hall and Ratledge, 1982 1984 1985; Barclay et al., 1985).

The aim of the current study was to provide an updated assessment of mycobacterial iron acquisition via siderophores. This was achieved by leveraging genomic data from respiratory and environmental Hawai’i NTM isolates (Honda et al., 2016; Virdi et al., 2021) as well as NTM genomic data from the NCBI GenBank public database. Mycobacterial iron-acquisition gene comparisons among the RGM and SGM and between respiratory and environmental isolates from Hawai’i were assessed. To further confirm siderophore production, *in vitro* microbiological tests were performed using the same respiratory and environmental isolates from Hawai’i under low iron culture conditions.

## 2. Materials and methods

### 2.1. NTM isolates used in this study

We collected 51 Hawai’i NTM isolates, including 28 respiratory and 23 environmental NTM isolates (Tables 1, 2) for analysis. Respiratory isolates were from de-identified individuals living in Hawai’i with suspected mycobacterial infections whose sputum had been submitted for mycobacterial culture and processed by the Diagnostic Laboratory Services, Inc (Aiea, HI). Environmental NTM isolates were collected by our group from indoor household water biofilms including household shower heads and sink faucets. A minority of water biofilms were collected from outdoor sources in Hawai’i including beach showerheads and garden hoses (Honda et al., 2016; Virdi et al., 2021). A description of isolate collection processes and DNA extraction methods are published (Honda et al., 2016; Virdi et al., 2021). All environmental isolates tested in this study were recovered from O’ahu, Hawai’i. Respiratory and environmental isolates were stratified as RGM or SGM. For the Hawai’i isolate panel tested, *M. abscessus* and *M. porcinum* were included as representative RGM. *M. intracellulare* subspecies *chimaera* (referred herein as *M. chimaera*) was used as a representative SGM. Ethical review and approval were waived because the clinical isolates used in this study were de-identified patient residual isolates and not considered human subject research.

**Table 1:** Respiratory NTM isolates used in this study.

	Isolate	ID used in phylogenetic trees	NCBI accession
1	248.MAB (MABS1)	MAB1	SAMN37352598
2	410.MAB (MAB2)	MAB2	SAMN37352599
3	423.MAB (MAB3)	MAB3	SAMN37352600
4	448.MAB (MAB4)	MAB4	SAMN37352601
5	528.MAB (MAB5)	MAB5	SAMN21208659
6	266.MAB (MAB6)	MAB6	SAMN21208657
7	311.MAB (MAB7)	MAB7	SAMN37352602
8	282.MPORC (MPORC1)	MPORC1	SAMN37352603
9	224.MPORC (MPORC2)	MPORC2	SAMN37352604
10	274.MPORC (MPORC3)	MPORC3	SAMN37352605
11	548.MPORC (MPORC4)	MPORC4	SAMN37352606
12	555.MPORC (MPORC5)	MPORC5	SAMN37352607
13	636.MPORC (MPORC6)	MPORC6	SAMN37352608
14	243.MPORC (MPORC7)	MPORC7	SAMN37352609
15	201.MCHIM (MCHIM1)	MCHIM1	SAMN37352610
16	202.MCHIM (MCHIM2)	MCHIM2	SAMN37352611
17	212.MCHIM (MCHIM3)	MCHIM3	SAMN37352612
18	225.MCHIM (MCHIM4)	MCHIM4	SAMN37352613
19	234.MCHIM (MCHIM5)	MCHIM5	SAMN37352614
20	242.MCHIM (MCHIM6)	MCHIM6	SAMN37352615
21	251.MCHIM (MCHIM7)	MCHIM7	SAMN37352616
22	252.MCHIM (MCHIM8)	MCHIM8	SAMN37352617
23	253.MCHIM (MCHIM9)	MCHIM9	SAMN37352618
24	254.MCHIM (MCHIM10)	MCHIM10	SAMN37352619
25	335.MCHIM (MCHIM11)	MCHIM11	SAMN37352620
26	406.MCHIM (MCHIM12)	MCHIM12	SAMN37352621
27	534.MCHIM (MCHIM13)	MCHIM13	SAMN37352622
28	733.MCHIM (MCHIM14)	MCHIM14	SAMN37352623

MAB, *Mycobacterium abscessus*; MPORC, *Mycobacterium porcinum*; MCHIM, *Mycobacterium chimaera*.

**Table 2:** Environmental NTM isolates used in this study.

	Isolate	ID used in phylogenetic trees	Source	NCBI accession
1	12-39-Sw-B-1.MAB (MAB8)	MAB8	Kitchen-sink	SAMN21208626
2	12-39-SW-B-2.MAB (MAB9)	MAB9	Kitchen-sink	SAMN37352124
3	12-45-Sw-A-2.MAB (MAB10)	MAB10	Kitchen-sink	SAMN37352125
4	12-9-SW-B-2.MAB (MAB 11)	MAB11	Showerhead	SAMN21208670
5	17-15-Sw-A1-1-37.MAB (MAB12)	MAB12	Sink	SAMN37352126
6	17-17-Sw-A1-1-37.MAB (MAB13)	MAB13	Showerhead	SAMN37352127
7	17-51-Sw-B1-1-37.MAB (MAB14)	MAB14	Kitchen-sink	SAMN37352128
8	17 N-17-Sw-B1-1-37.MAB (MAB15)	MAB15	Showerhead	SAMN37352129
9	17-101-Sw-C1-1.MPORC (MPORC8)	MPORC8	Showerhead	SAMN37352130
10	17-17-Sw-B1-1-37.MPORC (MPORC9)	MPORC9	Garden-Hose	SAMN37352131
11	17 N-17-Sw-B1-2-37.MPORC (MPORC10)	MPORC10	Showerhead	SAMN37352132
12	18-204-Sw-A1-1-37.MPORC (MPORC11)	MPORC11	Sink	SAMN37352133
13	17-9-Sw-A1-1-37.MPORC (MPORC12)	MPORC12	Beach-Showerhead	SAMN37352134
14	17 N-17-Sw-E1-37.MPORC (MPORC13)	MPORC13	Garden-Hose	SAMN37352135
15	17-54-Sw-A-1-1-30.MCHIM (MCHIM16)	MCHIM16	Sink	SAMN37352136
16	17-65-Sw-A1-1-37.MCHIM (MCHIM17)	MCHIM17	Kitchen-sink	SAMN37352137
17	17-65-Sw-B1-1-37.MCHIM (MCHIM18)	MCHIM18	Showerhead	SAMN37352138
18	KM16-1-Sw-1-30.MCHIM (MCHIM19)	MCHIM19	NP *	SAMN37352139
19	KM16-15-Sw-2-30.MCHIM (MCHIM20)	MCHIM20	Toilet	SAMN37352140
20	KM16-16-Sw-1-37.MCHIM (MCHIM21)	MCHIM21	Sink	SAMN37352141
21	KM16-20-Sw-1-30.MCHIM (MCHIM22)	MCHIM22	Sink	SAMN37352142
22	KM16-33-Sw-2-30.MCHIM (MCHIM23)	MCHIM23	Sink	SAMN37352143
23	KM16-9-Sw-1-30.MCHIM (MCHIM24)	MCHIM24	Sink	SAMN37352144

MAB, *Mycobacterium abscessus*; MPORC, *Mycobacterium porcinum*; MCHIM, *Mycobacterium chimaera*.

### 2.2. Whole-genome sequencing

DNA isolation for Illumina whole-genome sequencing (WGS) and genome assembly were performed as previously described (Epperson and Strong, 2020; Hasan et al., 2021). To contextualize the Hawai’i isolate data, DNA sequences for 47 additional non-Hawai’i respiratory and environmental NTM were downloaded from the publicly available National Center for Biotechnology Information (NCBI) GenBank database and are referred in this study as “NTM reference” isolates (Supplementary Tables 1A,B). Reference isolates used were *M. abscessus* subsp. *abscessus* (*n* = 1)*, M. abscessus* subsp. *massiliense* (*n* = 2)*, M. abscessus* subsp. *bolletii* (*n* = 1), *M. avium* (*n* = 5)*, M. intracellulare* (*n* = 4)*, M. paraintracellulare* (*n* = 1)*, M. chimaera* (*n* = 1)*, M. marseillense* (*n* = 1)*, M. sinensis* (*n* = 1)*, M. terrae* (*n* = 1), and *M. kansasii* (*n* = 1). There was no reference available for *M. porcinum*.

### 2.3. Siderophore gene analyses

The Kyoto Encyclopedia of Genes and Genomes (KEGG) database (Kanehisa et al., 2019) was used to interrogate NTM siderophore genes. Genes related to iron acquisition were identified by utilizing the KEGG pathway database to generate a list of each gene in the *mbt, mmpL/S*, and *esx-*3 gene pathways for each NTM species. All gene sequences with “*mbt*” in the gene name were acquired through the KEGG database.

Control metrics were performed to ensure the quality of the genome assemblies for each species: genome length (Supplementary Figure 1A), number of contigs (Supplementary Figure 1B), and the number of annotated genes (Supplementary Figure 1C). To assess the presence or absence of mycobactin (*mbt*), *mmpL/S*, and *esx-3* genes, WGS were annotated using Prokka (Seemann, 2014) and compiled into a pan-genome using Roary (Page et al., 2015). The genomes of respiratory and environmental Hawai’i NTM were parsed for *mbt*, *mmpL*, and *esx-3* genes, and the sequences of each gene for each isolate were aligned in a multiple sequence alignment using Geneious Prime (Version 2020.1).^1^ For each gene, phylogenetic trees were created to investigate genetic variation between species and between respiratory and environmental isolates within each species as well.

### 2.4. *In vitro* siderophore screening

Siderophore assays were adapted from prior work by Hall and Ratledge (1982) and Louden et al. (2011). The NTM Hawai’i panel (Tables 1, 2) was streaked for isolation onto Middlebrook 7H10 OADC (oleic acid albumin dextrose catalase) plates and incubated at 37°C for 7 days for RGM and up to 21 days for *M. chimaera* to generate stock cultures. From stock cultures, isolates were picked and streaked for isolation onto low iron culture media (glycerol, L-asparagine, potassium phosphate monobasic, zinc chloride, manganese (II) chloride, magnesium sulfate heptahydrate) with agar. Low iron plates were incubated at 37°C for 9–16 days for RGM and up to 28 days for *M. chimaera*. Growth on low iron agar plates was indicative of viable bacteria and siderophore production. No growth on low iron plates was recorded as no siderophore production. In parallel, low iron culture broth was inoculated with the same panel of NTM isolates and incubated for 1 month. The chrome azurol S (CAS) assay was used to detect siderophore activity and adapted from Louden et al. (2011). 1 mL of low iron NTM cultures were dispensed into wells of a 24 well plate with 100 μL of CAS reagent per the Louden protocol. Samples were incubated at room temperature for 15 min. Siderophore activity was noted by an eye-visible color change from blue to yellow/orange 24 h post inoculation. The results of the CAS test and the low iron media plates were used to conclude which isolates produced siderophores in low iron environments. *Mycobacterium neoaurum* and *Mycobacterium fortuitum* were, respectively, used as positive and negative controls for the CAS assay.

### 2.5. Statistical analyses

Statistical tests were done in R Studio and Prism. The One Sample Student’s t-test was used to compare the group with a higher or lesser number of genes between respiratory and environmental groups in the KEGG dataset. The Mann–Whitney-Wilcoxon test was used to determine whether the number of genes of respiratory and environmental groups for the Hawai’i data set were identical. The Kruskal-Wallis test was used to compare the number of genes in the comparison of respiratory/environmental and RGM/SGM from KEGG and to compare the number of genes between *M. abscessus, M. chimaera,* and *M. porcinum* from Hawai’i. The Games-Howell nonparametric post-hoc test was used to further compare the number of genes in the comparison of respiratory/environmental and RGM/SGM from different pairings of each group from KEGG. The Fisher’s exact test was used to compare siderophore growth presence in RGM/SGM and respiratory/environmental samples for each *in vitro* analysis due to the small sample size.

## 3. Results

### 3.1. *mbt* genes are more abundant among respiratory NTM isolates compared to environmental NTM isolates

**Figure 1:**
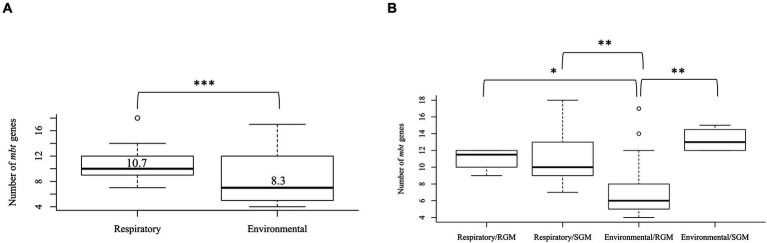
Varying number of mycobactin (*mbt*) genes between respiratory and environmental reference NTM isolates (Supplementary Tables 1A, B). Box plot showing **(A)** the number of *mbt* genes stratified by respiratory vs. environmental derived NTM; ***p = 0.0001989. **(B)** Number of *mbt* genes for respiratory vs. environmental NTM stratified by rapid growing mycobacteria (RGM) and slow growing mycobacteria (SGM). The central rectangle of the plot spans the interquartile range (IQR). The bar inside the rectangle represents the median, and the bars above and below show the location of the maximum and minimum, respectively. The small circle represents outliers. *p < 0.05; **p < 0.005.

The *mbt* genes available through KEGG were identified from 47 NTM isolates including 20 respiratory NTM, 27 environmental NTM, and two species from the *Mtb* complex for comparison (*Mtb* H37Rv and *M. canetti*). Overall, respiratory NTM isolates showed significantly more *mbt* genes (*n* = 10.7 genes) compared to environmental NTM (*n* = 8.3 genes) (*p* = 0.0002) (Figure 1A). When the respiratory and environmental isolates were stratified by RGM and SGM, the mean number of *mbt* genes was statistically different between respiratory/RGM and environmental/RGM group (*p* < 0.05), the respiratory/SGM and environmental/RGM group (*p* < 0.005), and the environmental/RGM and environmental/SGM group (*p* < 0.005) (Figure 1B).

### 3.2. *Mycobacterium chimaera* shows more *mbt* genes compared to *M. abscessus* or *M. porcinum*

**Figure 2:**
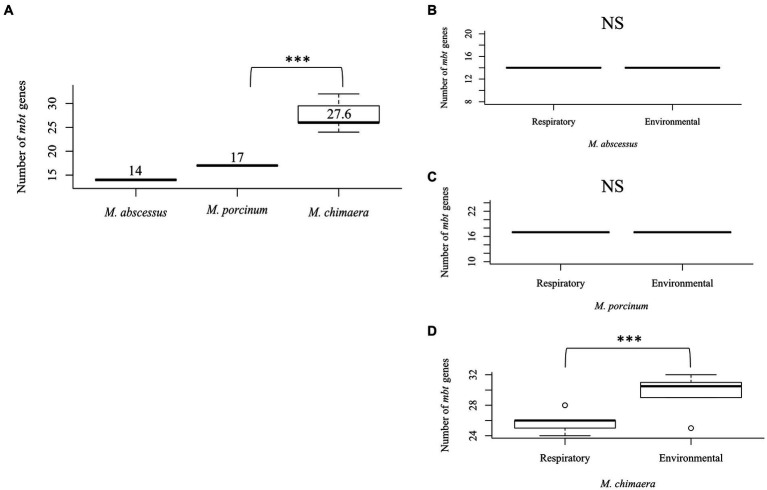
*Mycobacterium chimaera* shows more *mbt* genes compared to *M. abscessus* and *M. porcinum* **(A)** Distribution of the number of *mbt* genes among all *M. abscessus*, *M. chimaera*, and *M. porcinum* isolates from Hawai’i. ***p = 0.0005097. Number of *mbt* genes between respiratory or environmental **(B)** *M. abscessus*, **(C)** *M. porcinum*, **(D)** *M. chimaera*. ***p = 0.0005097. NS, not significant. Isolates studied in Figure 2 are listed in Tables 1 2.

To study the differences between the number of *mbt* genes among NTM species that can cause respiratory disease, 51 isolates were compared including seven respiratory and eight environmental isolates of *M. abscessus,* seven respiratory and six environmental isolates of *M. porcinum*, and 14 respiratory and nine environmental isolates of *M. chimaera*. Comparing both respiratory and environmental isolates, gene duplications were observed for *mbtC*, *mbtD*, *mbtE*, and *mbtF*, generally located outside of the *mbt* locus (average: 888,213 bp; range: [1,328:2,671,854 bp]), and varied by gene and species. Among the RGM *M. abscessus* and *M. porcinum* isolates, only one *mbtD* gene was identified. *M. abscessus* showed an average number of 14 *mbt* genes while *M. porcinum* showed an average of 17 genes, which was significantly less than the 27 *mbt* genes for *M. chimaera* (*p* = 0.005) (Figure 2A). Of note, the *mbt-2* gene complex, *mbtL*, *mbtM*, and *mbtN* genes were absent from *M. abscessus* and *mbtI* and *mbtL* genes were missing from *M. porcinum.* All ten *mbt-1* and four *mbt-2* genes were detected in *M. chimaera* accounting for its significantly higher average number of *mbt* genes.

Similar numbers of *mbt* genes were observed between respiratory and environmental *M. abscessus* (Figure 2B) and respiratory and environmental *M. porcinum* (Figure 2C) isolates. However, respiratory *M. chimaera* isolates showed significantly lower numbers of *mbt* genes compared to environmental *M. chimaera* isolates (*p* = 0.005) (Figure 2D).

**Figure 3:**
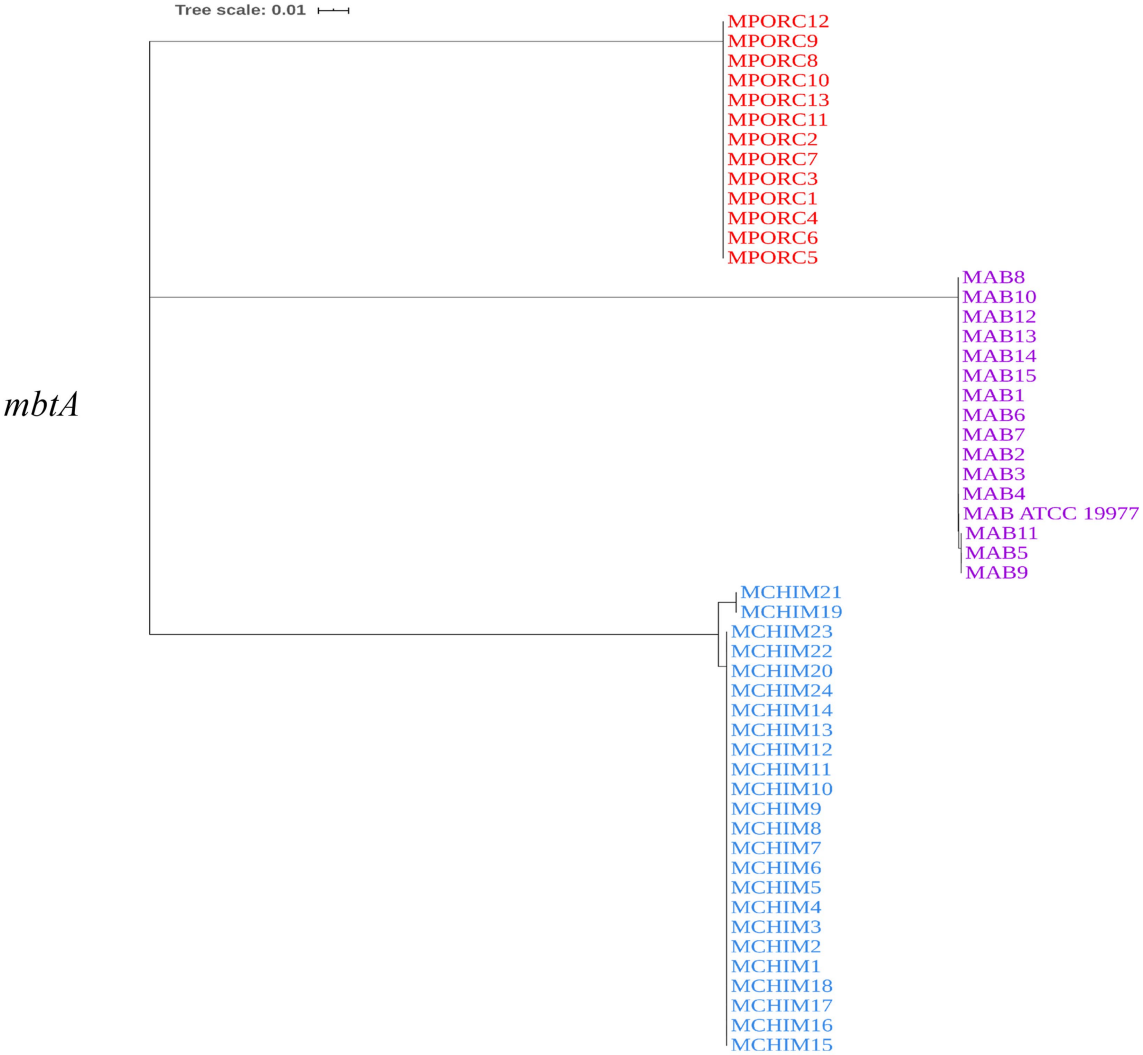
*mbtA* is genetically similar among the NTM. Neighbor-joining phylogenetic tree based on the *mbtA* gene. Scale bar indicates number of substitutions per site.

We constructed a phylogenetic tree to examine the genetic relatedness of *mbtA* genes among *M. abscessus, M. porcinum,* and *M. chimaera.* Each isolate contained one copy of the *mbtA* gene. *M. abscessus mbtA* is genetically similar to *M. porcinum mbtA* genes, as revealed by the close proximity in location on the phylogenetic tree (Figure 3). All sequences from respiratory and environmental isolates of *M. porcinum* were identical. This was also observed for *M. abscessus,* except for one respiratory and two environmental isolates which shared a single nucleotide polymorphism (SNP). For *M. chimaera*, two environmental isolates showed the same 13 SNPs while the rest of the samples were identical in sequence. Similar results can be seen for the mbt synthase G, *mbtG* gene (Supplementary Figure 2), where respiratory and environmental isolates within *M. abscessus* and *M. porcinum* and had identical gene sequences compared to two sub-branches for the *M. chimaera* cluster.

For the mycobactin synthases *mbtM* gene, two copies of the gene were identified in *M. porcinum* and *M. chimaera*. All *M. porcinum* isolates had identical sequences for both *mbtM* copies. For one copy of *mbtM* in *M. chimaera,* the two environmental isolates sharing the *mbtA* variant also shared an *mbtM* copy variant with 18 SNPs. The other *mbtM* copy had the same relationships as within the *mbtG* gene of *M. chimaera*. This pattern of relationships was also observed in the *mbtM* and *mbtN* genes in *M. porcinum* and *M. chimaera*; however, these genes are single-copy in all isolates (Supplementary Figures 3A,B).

### 3.3. *mmpl*/S and *esx*-3 secretion system associated genes in NTM from Hawai’i

Because of their roles in siderophore and iron transport, we also investigated the number of *mmpL/S* and *esx*-3 secretion system associated genes in the same sample of Hawai’i NTM isolates. There were 13 *mmpL* proteins based on a model of *Mtb*, but we focused on *mmpL/S3, mmpL/S4,* and *mmpL/S5* for their roles in either siderophore or heme export (Szekely and Cole, 2016). While the number of *mmpL345* and *mmpS345* genes tended to follow the trend of *M. abscessus* > *M. porcinum* > *M. chimaera*, no significant differences were observed (Supplementary Figures 4A,B). When the number of *mmpL* genes were compared between respiratory or environmental isolates among the three different species, no significant differences were found (Supplementary Figures 5A–C).

**Figure 4:**
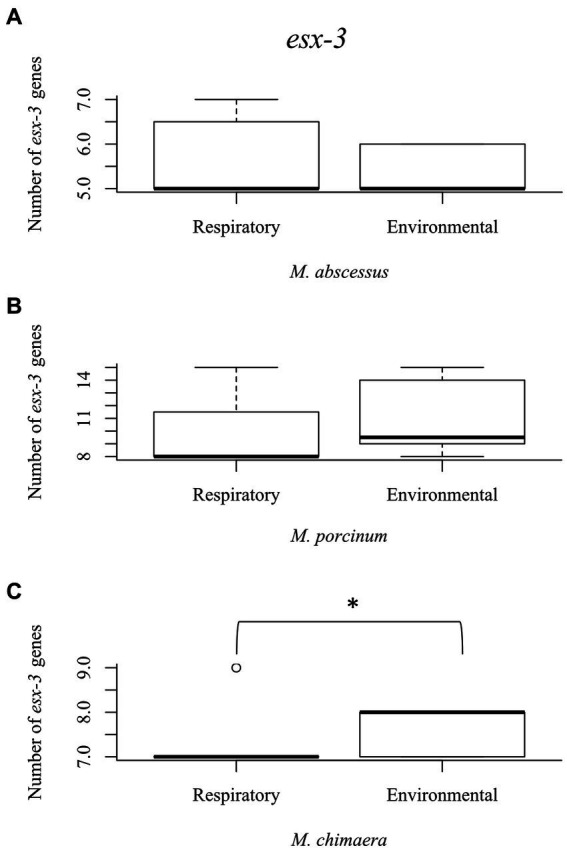
*Mycobacterium chimaera* shows significantly more *esx-3* genes compared to rapid growing mycobacteria. Box plot showing the number of *esx*-3 genes in respiratory and environmental **(A)** *M. abscessus;* **(B)** *M. porcinum;* and **(C)** *M. chimaera*. *p = 0.01346.

The esx-3 secretion system is required for mycobacterial iron acquisition through *mbt* (Siegrist et al., 2009). The *esx*-3 locus contains the genes for the *esx*G and *esx*H proteins, and supporting genes identified as *ecc*A3, *ecc*B3, *ecc*C3, *ecc*D3, eccE3*, esp*G3, and *myc*P3 that are required for protein export and iron acquisition (Siegrist et al., 2014). While *esx*-3 gene numbers did not vary among the RGM isolates (Figures 4A,B), environmental *M. chimaera* isolates showed significantly more numbers of *esx*-3 genes compared to respiratory *M. chimaera* isolates (*p* = 0.013) which appear to be due to gene duplications of the *mycP3* gene (Figure 4C). Among Hawai’i *M. chimaera*, we found 1–3 copies of *esp*G3 or *mycP*3 per genome. The duplicate copies of *esp*G3 and *mycP*3 were inconsistently located throughout the genome. The average distance between *esp*G3 copies was 1,979,955 nt (range: 560,978–2,891,545 nt) and the average distance between *mycP*3 copies was 2,636,478 nt (range: 479,373 – 4,820,547 nt).

### 3.4. *In vitro* siderophore production is higher in RGM than in SGM

Next, two *in vitro* methodologies, including growth on low iron media plates and the CAS detection assay, were used to screen the isolates for siderophore production in low iron environments and results are summarized in Supplementary Tables 3, 4. Of the environmental *M. abscessus, M. porcinum,* and *M. chimaera* isolates tested, 88% (*n* = 7/8), 100% (*n* = 6/6), and 11% (*n* = 1/9), respectively showed visible colonies indicative of growth on low iron plates. Of the respiratory *M. abscessus*, *M. porcinum*, and *M. chimaera* isolates tested, 100% (*n* = 7/7), 86% (*n* = 6/7), and 29% (*n* = 4/14), respectively showed visible colonies indicative of growth on low iron plates.

**Figure 5:**
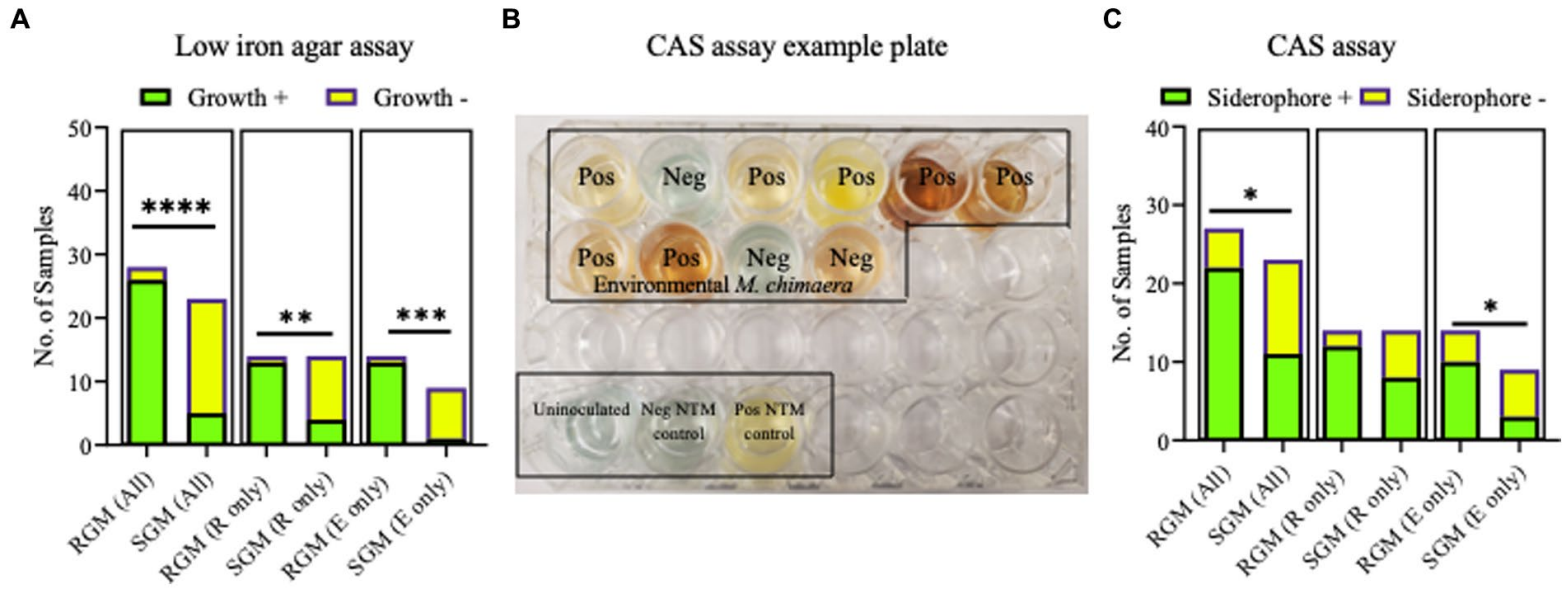
Growth on low iron agar and positive CAS assays are more often observed for rapid growing mycobacteria (RGM) compared to slow growing mycobacteria (SGM). **(A)** Stacked bar graphs showing +/− growth for RGM and SGM on low iron agar plates. R, respiratory derived isolates. E, environment derived isolates. Low iron RGM vs SGM ****p = <0.0001, ***p = 0.0002, *p = 0.0175. **(B)** To demonstrate CAS assay outcomes, we show an example of positive and negative media color change results for 10 environmental *M. chimaera* isolates, black box. *M. neoaurum* and *M. fortuitum* were used as positive and negative control isolates, respectively along with an uninoculated well (bottom of image, black box). **(C)** Outcomes for all CAS assays shown as stacked bar graphs. *R*, respiratory derived isolates. E, environment-derived isolates. *p < 0.05.

No differences were observed between respiratory and environmental NTM growth on low iron plates (*p* > 0.9999) (data not shown). When these data were stratified by RGM (*M. abscessus* and *M. porcinum*) and SGM *(M. chimaera)* categories, growth on low iron agar was more frequently observed for the RGM compared to the SGM (*p* < 0.0001) (Figure 5A, left panel). This trend was also true when the isolates were stratified as respiratory RGM and SGM isolates (*p* = 0.018) (Figure 5A, middle panel) and environmental RGM and SGM isolates (*p* = 0.0002) (Figure 5A, right panel). No statistical differences were observed between the number of environmental and respiratory isolates that grew or did not grow on low iron agar.

The universal siderophore assay, CAS, was used as a second assay to assess siderophore production by NTM isolates. When a strong iron chelator such as a siderophore removes iron from the media a color change from green/blue to yellow/orange occurs. In Figure 5B, we show an example of CAS assay outcomes using 10 environmental *M. chimaera* isolates. Eight isolates (80%) showed a yellow/orange color change (positive) and two (20%) showed a green/blue color indicative of a negative test. *M. neoaurum* and *M. fortuitum* were used as positive and negative controls, respectively along with an uninoculated well. Finally, we tested the collection of environmental and respiratory NTM by the CAS assay. Among environmental *M. abscessus, M. porcinum,* and *M. chimaera* isolates, 88% (*n* = 7/8), 50% (*n* = 3/6), and 30% (*n* = 3/10), respectively showed positive color change for CAS. Of the respiratory *M. abscessus, M. porcinum,* and *M. chimaera* isolates tested, 100% (*n* = 7/7), 71% (*n* = 5/7), and 57% (n = 8/14), respectively showed positive color change for CAS (Supplementary Tables 3, 4). We did not observe any significant difference in CAS outcomes between environmental and respiratory isolate groups or when the isolates were sub-categorized by species. We observed more CAS positive tests in RGM versus SGM (*p* < 0.05) (Figure 5C, left panel) and among environmental isolates (e.g., environmental RGM vs. environmental SGM) (*p* < 0.05) (Figure 5C, middle panel), but not when comparing within respiratory isolates (e.g., respiratory RGM vs. respiratory SGM) (Figure 5C, right panel).

Because all isolates studied were tested for siderophore activity *via* growth on low iron plates and by the CAS assay, we determined the frequency at which the outcomes of the two assays matched or mismatched. We highlight in red font the between assay matches per isolate tested in Supplementary Tables 1A,B. Overall, among all 28 RGM isolates tested, 22 (79%) showed low iron assay outcomes matched CAS assay outcomes compared to 21% (6/28) mismatches (Supplementary Figure 6). When analyzed separately, assay concurrency was more often observed for respiratory isolates compared to environmental RGM isolates (93% vs. 64%) (Supplementary Figure 7). Taking an additional step, we separated the outcomes for *M. abscessus* and *M. porcinum.* Generally, low iron assay results aligned with CAS assay outcomes for respiratory *M. abscessus* (7/7) and *M. porcinum* (6/7) isolates, agreeing 100 and 86%, respectively, of the time. Among the environmental *M. abscessus* and *M. porcinum* isolates tested, the two assay outcomes agreed 75% (6/8) and 50% (3/6), respectively.

Similarly, for the SGM, 70% (16/23) *M. chimaera* isolates studied showed low iron assay outcomes matched CAS assay outcomes compared to 30% (7/23) mismatches (Supplementary Figure 6). By stratifying all *M. chimaera* isolates into respiratory and environmental categories, concurrency between the two tests was more often observed for environmental *M. chimaera* isolates compared to respiratory *M. chimaera* isolates (89% vs. 57%) (Supplementary Figure 7).

**Figure 6:**
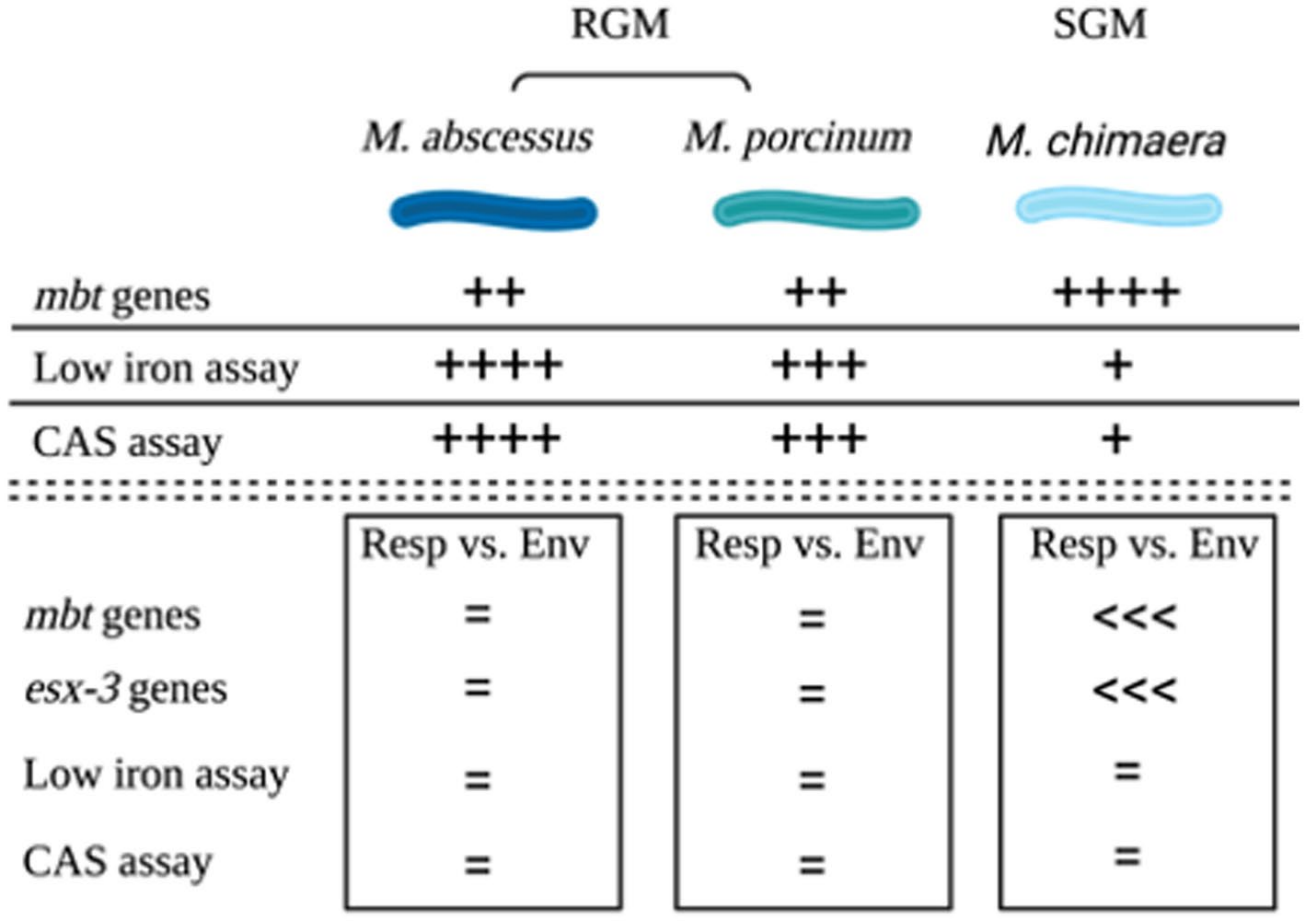
Summary. Cartoon summarizing the outcomes of genomic comparisons and applied microbiological assays to assess siderophores among *M. abscessus, M. porcinum*, and *M. chimaera* respiratory and environmental isolates. Image created using BioRender.

The combined outcomes from genomic analyses and *in vitro* assays for the three NTM species and subspecies tested in this study are summarized in a cartoon, Figure 6.

## 4. Discussion

NTM PD is a growing cause of concern as its prevalence has been increasing in Hawai’i, nationwide, and globally. A panel of NTM from Hawai’i comprised of environmental and respiratory isolates was used to examine differences, if any, between *M. abscessus, M. porcinum*, and *M. chimaera* isolates in terms of iron acquisition by siderophores when stratified by their (1) recovered niche and (2) growth type (i.e., RGM or SGM).

In the U.S., the mean amount of iron in surface soil (0–5 centimeters) is 214 μg/L or 0.214 mg/kg (Smith et al., 2013; Brittenham et al., 2018). For comparison, this is lower than the mean amount of iron in Hawaiian Island soils that is around 95,500 mg/kg (Hawai’i Department of Health Hazard Evaluation and Emergency Response, 2012). Drinking water in the U.S. seldom contains more than 300 μg/L (Services). The normal range for serum iron is 6 to 17 μg/L and iron is present in the cytoplasm of host cells at very low levels (Agoro and Mura, 2019). In prolonged iron starvation environments such as the granuloma, *Mtb* can show high expression of *mbt* genes and siderophore production (Agoro and Mura, 2019). Due to the limited amount of iron in the body compared to the overabundance of iron in the extracellular niches that environmental NTM inhabit, we hypothesized that respiratory NTM would be more likely to produce siderophores due to the limited access to iron in the lung, compared to environmental NTM isolates. To test this hypothesis, we performed complementary genomic and microbiological analyses to assess siderophore production.

Among the reference NTM gene sequences parsed through the KEGG database that we examined, respiratory NTM showed significantly higher mean number of *mbt* genes compared to environmentally isolated species (Figure 1A). On the other hand, the number of *mbt* genes were similar among the respiratory and environmental NTM isolates from Hawai’i based on the phylogenetic trees and multiple sequence alignments (Figure 3; Supplementary Figures 2, 3). For the Hawai’i isolates, we detected all ten *mbt-1* and four *mbt-2* genes in SGM *M. chimaera* compared to the RGM species (containing nine to ten *mbt-1* and one to three *mbt-2* genes) suggesting *M. chimaera* is selectively advantaged in iron acquisition from the environment compared to *M. abscessus* and *M. porcinum.* When we further analyzed for the presence of *mbt* genes where each species was stratified by their recovered niches, there was no statistical difference in *mbt* gene numbers between the respiratory and environmental RGM isolates of *M. abscessus* and *M. porcinum* (Figures 2B,C). However, for SGM *M. chimaera* from Hawai’i, environmental isolates showed more *mbt* genes compared to respiratory isolates (Figure 2D). However, despite differences in the detection of siderophore genes among the isolates tested, we did not observe significant phenotypic differences in the *in vitro* assay outcomes.

Besides *mbt,* we also examined two other iron acquiring systems for mycobacteria, *mmpL*/S and *esx*-3. *mmpL3*, plays a key role in the acquisition of iron from heme (Tullius et al., 2011). *mmpL4* and *mmpL5* both have similar functions in iron acquisition *via* siderophores (Wells et al., 2013). In contrast to *mbt* genes of the SGM, the total number of *mmp*L proteins are more abundant in RGM compared to SGM, especially in the *M. abscessus* species (Viljoen et al., 2017). In our study, although not statistically significant, the number of *mmpL*/S genes tended to be higher in RGM compared to SGM (Supplementary Figure 4). The abundance of *mmpL* genes could allow the RGM to persist in various environments, and *M. abscessus* has been observed to evolve and acquire genetic material rapidly (Sapriel et al., 2016). Although, it is unknown how *esx*-3 plays a role in iron acquisition, it is essential for mycobacterial growth in iron-limited conditions. Similar to *mbt*, the environmental *M. chimaera* isolates showed significantly more esx-3 genes than respiratory *M. chimaera* isolates (Figure 4C). These genetic results support siderophore producing advantages among Hawai’i *M. chimaera* environmental isolates that likely facilitate survival in exogenous niches. *M. chimaera* was the most common NTM recovered from the environment of Hawai’i (Honda et al., 2016); thus, this subspecies may have evolved methods to procure iron in nutrient limited environmental conditions. We have already shown that *M. chimaera*-derived from the Hawai’i environment grows better in the presence of iron oxide minerals such as hematite after 24 h in culture and binds to its surface (Glickman et al., 2020).

To complement our genomic analysis, we tested our NTM Hawai’i isolate panel for siderophore production by two microbiological assays, growth on low iron culture media and the CAS assay. Such assays have already been applied to study siderophore production in a variety of gram-negative and gram-positive bacteria (Hall and Ratledge, 1982; Louden et al., 2011; Ferreira et al., 2019). Based on the results from our genomic analyses, we posited that siderophore production would be more frequently observed *in vitro* for *M. chimaera* isolates compared to *M. abscessus* and *M. porcinum* isolates. However, iron scavenging from culture media containing low amounts of iron and production of siderophores was more frequently observed for the RGM *M. abscessus* and *M. porcinum* isolates compared to SGM *M. chimaera* categories (Figures 5A,C). We also observed discrepancies across the outcomes for the microbiological assays; that is, siderophore test outcomes did not match 100% of the time. For example, for 22 of the 28 RGM isolates tested and for 16 of 23 SGM isolates tested, low iron assay results matched CAS assay results 79 and 70% of the time, leaving mismatch outcomes for 21 and 30% of the isolates tested (Supplementary Figure 6). The reasons for the incongruency between the genomic analyses and *in vitro* outcomes and between the two microbiological tests used in our study are not known. Other studies have demonstrated SGM show significantly more inorganic ion transport gene clusters than RGM (Bachmann et al., 2019). Alternatively, discrepancies may be related to innate adaptation to exogenous iron or differences in siderophore secretion as our *in vitro* assays detect secreted siderophores. There may also be features of each culture media (e.g., dextrose, glycerol, or other components) that may influence siderophore activity which may also vary per isolate and between isolates of different growth rates (e.g., RGM and SGM). Future studies to reassess NTM siderophore production as supplemental iron is added to culture media, utilizing similar media components per assay, and repeating low iron tests using liquid culture would help to disentangle some of these outcomes.

This study has some limitations. For the gene sequences taken through the KEGG database, there was limited information on some of the genomes. For instance, there is a lack of clear information on isolation details. The data from those NTM isolates were excluded from the dataset; thus, reducing the sample size and variety of isolates analyzed. Secondly, the genomes from Hawai’i were from bacteria isolated from the island of O’ahu. While O’ahu is home to the majority of the state’s population, it would be prudent to explore iron-acquisition mechanisms by NTM recovered from other islands and other geographic areas. In addition, the *in vitro* CAS assay analyses of extracellular siderophores was not included in the gene analysis of intracellular siderophores and other iron-acquisition processes. A follow up transcriptomic study would be helpful to explore other genes and proteins involved with iron metabolism (e.g., transferrin, ferritin) and discern ones that are modulated with low iron exposure.

To expand upon this study, future studies may include similar experiments to analyze extracellular siderophore genes and siderophore activity using a more diversified panel of SGM and RGM species with larger numbers of environmental and respiratory isolates to further verify differences among NTM. In addition, other factors affecting iron acquisition such as competition could be analyzed through a combined analysis of other NTM and non-NTM isolates. More importantly, the roles of *mbt, mmpL/S,* and *esx-3* in iron acquisition for *M. abscessus*, *M. porcinum*, and *M. chimaera* should be further evaluated for their roles in virulence in the context of lung immune cell infections, as has been performed for *Mtb* and *Salmonella* (De Voss et al., 2000; Saha et al., 2019).

In summary, our data provide new insights into iron acquisition genes and the presence and activity of NTM siderophores. Clinical studies should be performed to understand the role of dietary iron in individuals with NTM PD. Outcomes of these studies may highlight NTM iron acquisition genes or siderophores as new targets for drug development.

## Data availability statement

All relevant data is contained within the article. The original contributions presented in the study are included in the article/supplementary material, further inquiries can be directed to the corresponding author. Information for existing publicly accessible datasets is contained within the article. The datasets presented in this study can be found in online repositories. The names of the repository/repositories and accession number(s) can be found in the article/supplementary material. The data presented in this study are deposited in NCBI, accession numbers: SAMN37352598-SAMN37352623, SAMN21208657, SAMN21208659, SAMN37352124-SAMN37352144, SAMN21208626, SAMN212086270.

## Author contributions

CT: Data curation, Investigation, Methodology, Visualization, Writing – review & editing, Formal analysis, Software, Validation, Writing – original draft. NO: Data curation, Formal analysis, Investigation, Methodology, Validation, Visualization, Writing – review & editing. AM: Data curation, Formal analysis, Investigation, Validation, Visualization, Writing – review & editing. SD: Formal analysis, Investigation, Validation, Visualization, Writing – review & editing. YC: Writing – review & editing, Funding acquisition, Methodology, Project administration, Resources, Software, Supervision, Writing – original draft. MS: Methodology, Software, Supervision, Writing – review & editing. NH: Methodology, Software, Supervision, Writing – review & editing, Data curation, Formal analysis, Investigation, Validation, Visualization, Writing – original draft. JH: Data curation, Formal analysis, Investigation, Methodology, Supervision, Visualization, Writing – original draft, Writing – review & editing, Conceptualization, Funding acquisition, Project administration, Resources.

## Footnotes

^1^ https://www.geneious.com
