# Supplementary material for: Genomic and microbiological analyses of iron acquisition pathways among respiratory and environmental nontuberculous mycobacteria from Hawai’i
Source: Front Microbiol. 2023 Nov 10;14:1268963. doi: 10.3389/fmicb.2023.1268963 (PMC10667711; doi:10.3389/fmicb.2023.1268963)
Supplement: Supplementary file 1 [file Data_Sheet_1.PDF]

Supplementary Material

1 Supplementary Tables

Supplementary Table 1A. NTM Reference Respiratory Isolates used in this study from the KEGG Database (n=22)

Isolate	*GenBank Accession*
Mycobacterium canetti 140070010 (mcx)	FO203509
Mycobacterium tuberculosis H37Rv (mtu)	AL123456
Mycobacterium avium K-10 (mpa)	AE016958
Mycobacterium avium MAP4 (mao)	CP005928
Mycobacterium avium E1 (mavi)	CP010113
Mycobacterium avium E93 (mavu)	CP010114
Mycobacterium avium 104 (mav)	CP000479
Mycobacterium intracellulare MOTT (mit)	CP003323
Mycobacterium intracellulare ATCC 13950 (mia)	CP003322
Mycobacterium intracellulare (mid)	CP002275
Mycobacterium intracellulare subsp. yongonense (myo)	CP003347
Mycobacterium paraintracellulare (mir)	CP003324
Mycobacterium chimaera (mchi)	CP012885
Mycobacterium abscessus ATCC 19977 (mab)	CU458896
Mycobacterium abscessus subsp. massiliense GO 06 (mmv)	CP003699

Mycobacterium abscessus subsp. bolletii 50594 (mabb)	CP004374
Mycobacterium abscessus subsp. massiliense CCUG (mabl)	AP014547
Mycobacterium marseillense (mmal)	CP023147
Mycobacterium intracellulare sp. MOTT36Y (mmm)	CP003491
Mycobacterium sinensis (mjd)	CP002329
Mycobacterium terrae (mter)	LT906469
Mycobacterium kansasii ATCC (mkn)	CP006835

Supplementary Table 1B. NTM Reference Environmental Isolates used in this study from the KEGG Database (n=27)

Isolate	*GenBank Accession*
Mycobacterium stephanolepidis (mste)	AP018165
Mycobacterium goodii (mgo)	CP012150
Mycobacterium fortuitum (mft)	CP011269
Mycobacterium phlei (mphl)	CP014475
Mycobacterium vaccae (mvq)	CP011491
Mycobacterium ulcerans (mul)	CP000325
Mycobacterium sp MCS (mmc)	CP000384
Mycobacterium sp KMS (mkm)	CP000518
Mycobacterium sp JLS (mjl)	CP000580
Mycobacterium marinum M (mmi)	CP000854
Mycobacterium marinum E11 (mmae)	HG917972
Mycobacterium liflandii (mli)	CP003899
Mycobacterium sp VKM Ac-1817D (myv)	CP009914
Mycobacterium sp EPa45 (mye)	CP011773
Mycobacterium sp NRRL B-3805 (myn)	CP011022
Mycobacterium dioxanotrophicus (mdx)	CP020809
Mycobacterium smegmatis MC2 155 (msm)	CP000480
Mycobacterium smegmatis MC2 155 (msg)	CP001663

Mycobacterium smegmatis MC2 155 (msb)	CP009494
Mycobacterium smegmatis INHR1 (msn)	CP009495
Mycobacterium smegmatis INHR2 (msh)	CP009496
Mycobacterium vanbaalenii (mva)	CP000511
Mycobacterium gilvum PYR-GCK (mgi)	CP000656
Mycobacterium neoaurum (mne)	CP006936
Mycobacterium rhodesiae (mrh)	CP003169
Mycobacterium thermoresistable (mthn)	LT906469
Mycobacterium gilvum Spyr-1 (msp)	CP002385

Supplementary Table 3. Summarized outcomes for low iron and CAS assay for all RGM isolates

Red font indicates an isolate whose paired low iron and CAS assay results did not align

	Isolate	Respiratory or Environmental (R or E)	Low iron agar:	CAS assay:
1	248.MAB (MABS1)	R	Positive	Positive
2	410.MAB (MAB2)	R	Positive	Positive
3	423.MAB (MAB3)	R	Positive	Positive
4	448.MAB (MAB4)	R	Positive	Positive
5	528.MAB (MAB5)	R	Positive	Positive
6	266.MAB (MAB6)	R	Positive	Positive
7	311.MAB (MAB7)	R	Positive	Positive
8	282.MPORC (MPORC1)	R	Negative	Negative
9	224.MPORC (MPORC2)	R	Positive	Positive
10	274.MPORC (MPORC3)	R	Positive	Positive
11	548.MPORC (MPORC4)	R	Positive	Positive
12	555.MPORC (MPORC5)	R	Positive	Negative
13	636.MPORC (MPORC6)	R	Positive	Positive
14	243.MPORC (MPORC7)	R	Positive	Positive
OUTCOME:			13(+), 1(-)	12(+), 2(-)
1	12-39-Sw-B-1.MAB (MAB8)	E	Positive	Positive
2	12-39-SW-B-2.MAB (MAB9)	E	Positive	Positive
3	12-45-Sw-A-2.MAB (MAB10)	E	Positive	Negative
4	12-9-SW-B-2.MAB (MAB 11)	E	Negative	Positive
5	17-15-Sw-A1-1-37.MAB (MAB12)	E	Positive	Positive
6	17-17-Sw-A1-1-37.MAB (MAB13)	E	Positive	Positive
7	17-51-Sw-B1-1-37.MAB (MAB14)	E	Positive	Positive
8	17N-17-Sw-B1-1-37.MAB (MAB15)	E	Positive	Positive
9	17-101-Sw-C1-1.MPORC (MPORC8)	E	Positive	Positive

10	17-17-Sw-B1-1-37.MPORC (MPORC9)	E	Positive	Negative
11	17N-17-Sw-B1-2-37.MPORC (MPORC10)	E	Positive	Negative
12	18-204-Sw-A1-1-37.MPORC (MPORC11)	E	Positive	Positive
13	17-9-Sw-A1-1-37.MPORC (MPORC12)	E	Positive	Positive
14	17N-17-Sw-E1-37.MPORC (MPORC13)	E	Positive	Negative
OUTCOME:			13(+), 1(-)	10(+), 4(-)

Supplementary Table 4. Summarized outcomes for low iron and CAS assay for all SGM isolates

Red font indicates an isolate whose paired low iron and CAS assay results did not align

	Isolate	Respiratory or Environmental (R or E)	Low iron agar:	CAS assay:
1	201.MCHIM (MCHIM1)	R	Positive	Negative
2	202.MCHIM (MCHIM2)	R	Negative	Positive
3	212.MCHIM (MCHIM3)	R	Negative	Positive
4	225.MCHIM (MCHIM4)	R	Negative	Negative
5	234.MCHIM (MCHIM5)	R	Positive	Positive
6	242.MCHIM (MCHIM6)	R	Negative	Negative
7	251.MCHIM (MCHIM7)	R	Negative	Positive
8	252.MCHIM (MCHIM8)	R	Negative	Negative
9	253.MCHIM (MCHIM9)	R	Negative	Negative
10	254.MCHIM (MCHIM10)	R	Negative	Negative
11	335.MCHIM (MCHIM11)	R	Positive	Positive
12	406.MCHIM (MCHIM12)	R	Negative	Positive
13	534.MCHIM (MCHIM13)	R	Positive	Positive
14	733.MCHIM (MCHIM14)	R	Negative	Positive
OUTCOME:			4(+), 10(-)	8(+), 6(-)
1	17-54-Sw-A-1-1-30.MCHIM (MCHIM16)	E	Positive	Positive
2	17-65-Sw-A1-1-37.MCHIM (MCHIM17)	E	Negative	Positive
3	17-65-Sw-B1-1-37.MCHIM (MCHIM18)	E	Negative	Positive
4	KM16-1-Sw-1-30.MCHIM (MCHIM19)	E	Negative	Negative

5	KM16-15-Sw-2-30.MCHIM (MCHIM20)	E	Negative	Negative
6	KM16-16-Sw-1-37.MCHIM (MCHIM21)	E	Negative	Negative
7	KM16-20-Sw-1-30.MCHIM (MCHIM22)	E	Negative	Negative
8	KM16-33-Sw-2-30.MCHIM (MCHIM23)	E	Negative	Negative
9	KM16-9-Sw-1-30.MCHIM (MCHIM24)	E	Negative	Negative
OUTCOME:			1(+), 8(-)	3(+), 6(-)

2 Supplementary Figures

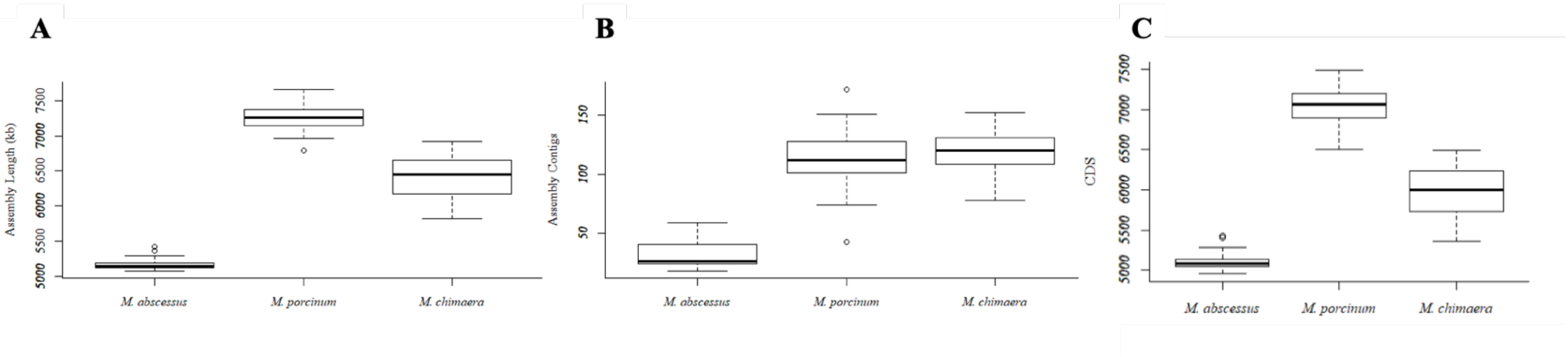

Supplementary Figure 1. Box plots showing the quality control metrics for each species. The central rectangle of the plot spans the interquartile range (IQR). The bar inside the rectangle represents the median, and the bars above and below show the location of the maximum and minimum, respectively. The small circles represent outliers. **A)** Assembly length. **B)** Number of assembly contigs. **C)** Number of CDS (annotated genes).

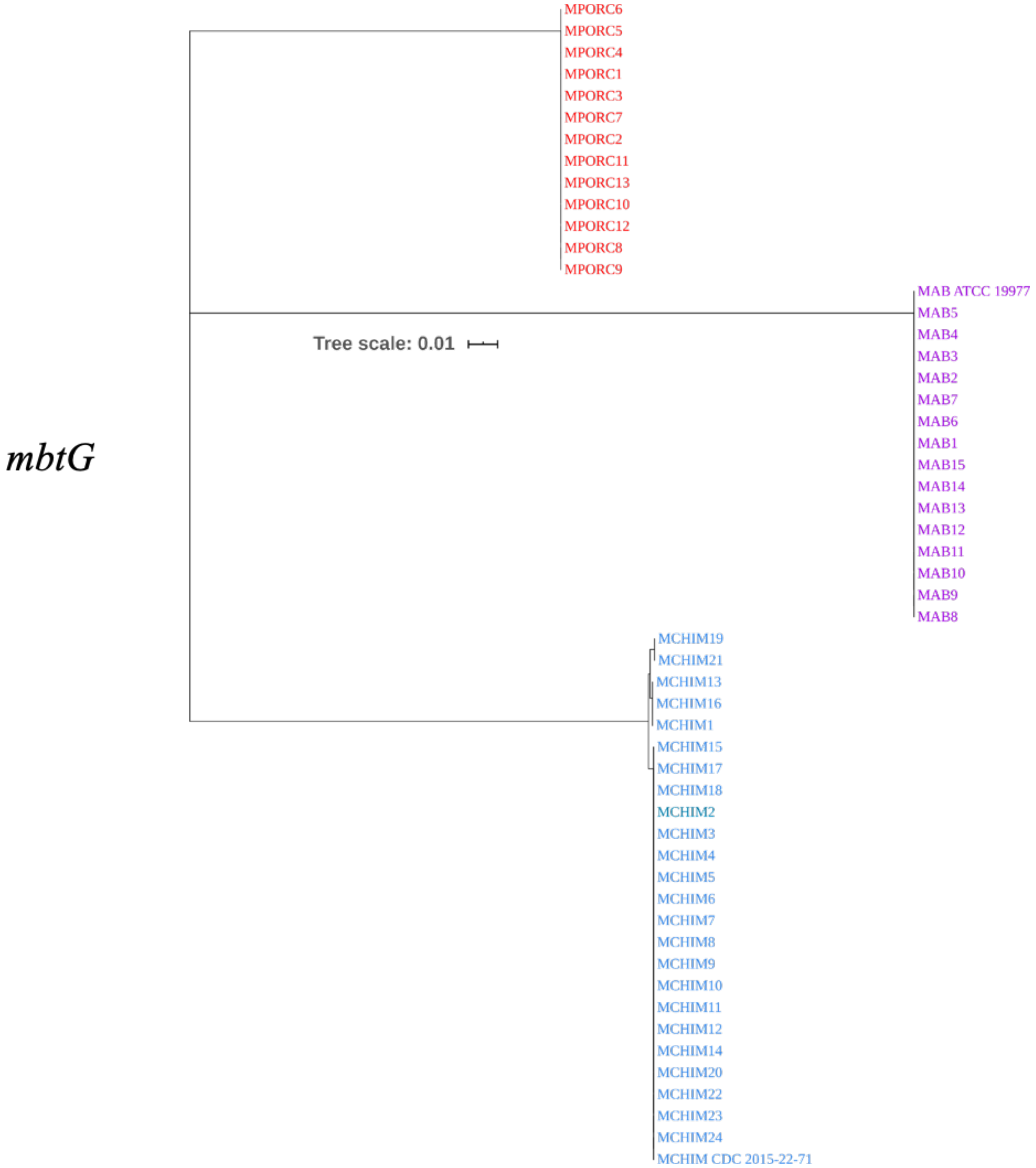

Supplementary Figure 2. *mbtG* is genetically similar among the NTM. Neighbor-joining phylogenetic tree based on the *mbtG* gene. Scale bar indicates number of substitutions per site.

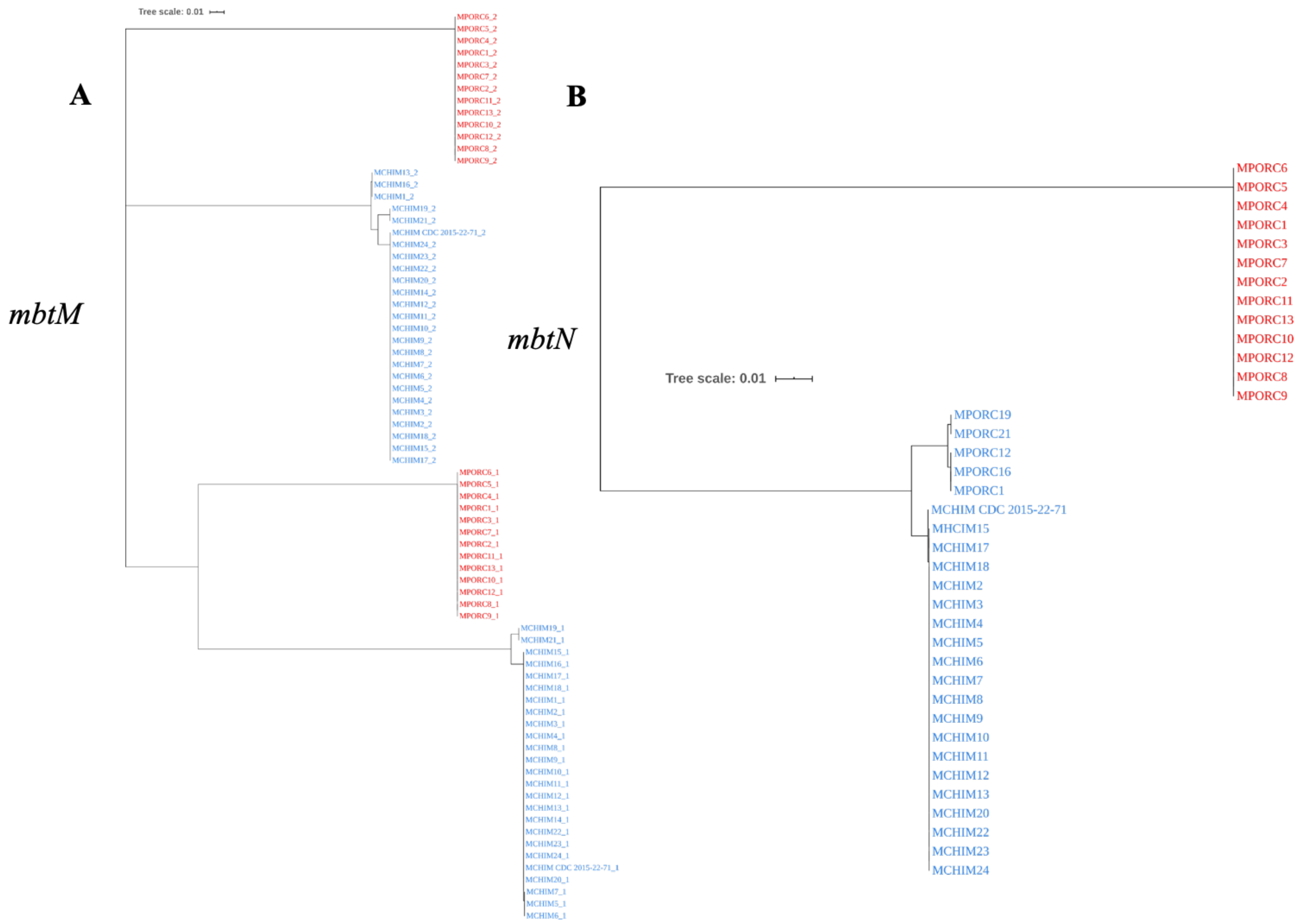

Supplementary Figure 3. Neighbor-joining phylogenetic tree based on the **A)** *mbtM* gene and **B)** *mbtN* gene. Scale bar indicates number of substitutions per site.

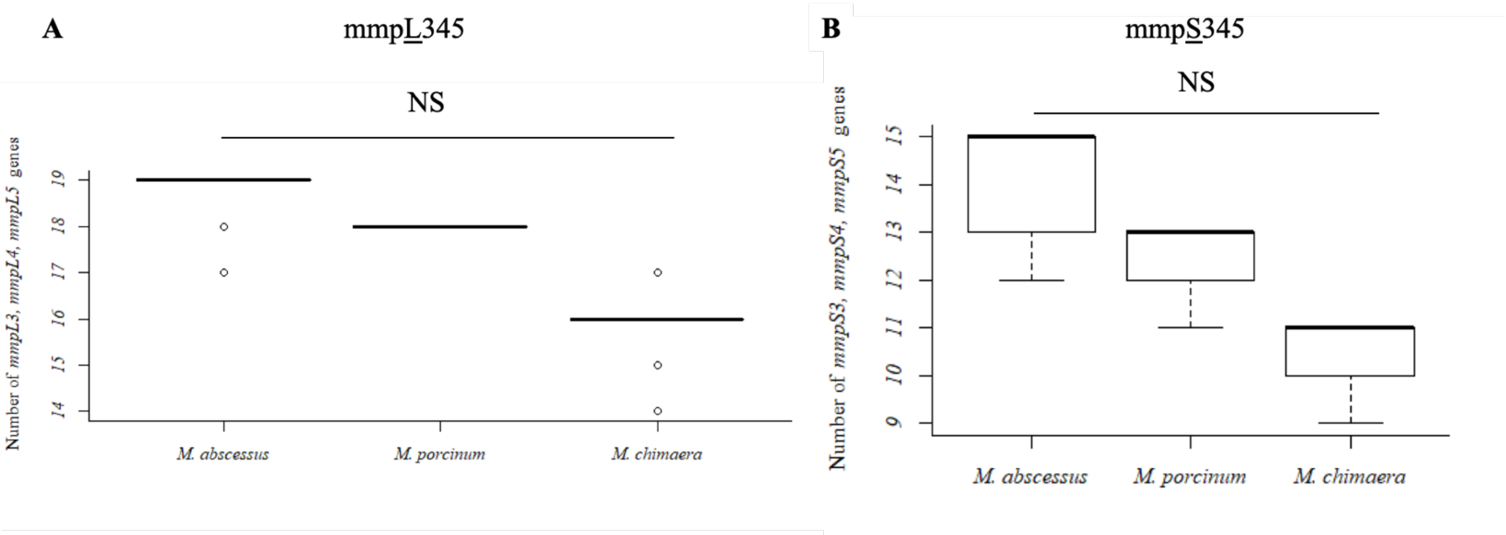

Supplementary Figure 4. Box plot showing the distribution of the number of **A)** *mmpL3, mmpL4, and mmpL5* genes and the number of **B)** *mmpS3, mmpS4, and mmpS5* genes in *M. abscessus*, *M. porcinum*, and *M. chimaera* from Hawai'i. Statistical significance was determined by using the Kruskal-Wallis Test with a p-value < 0.05. NS = not significant.

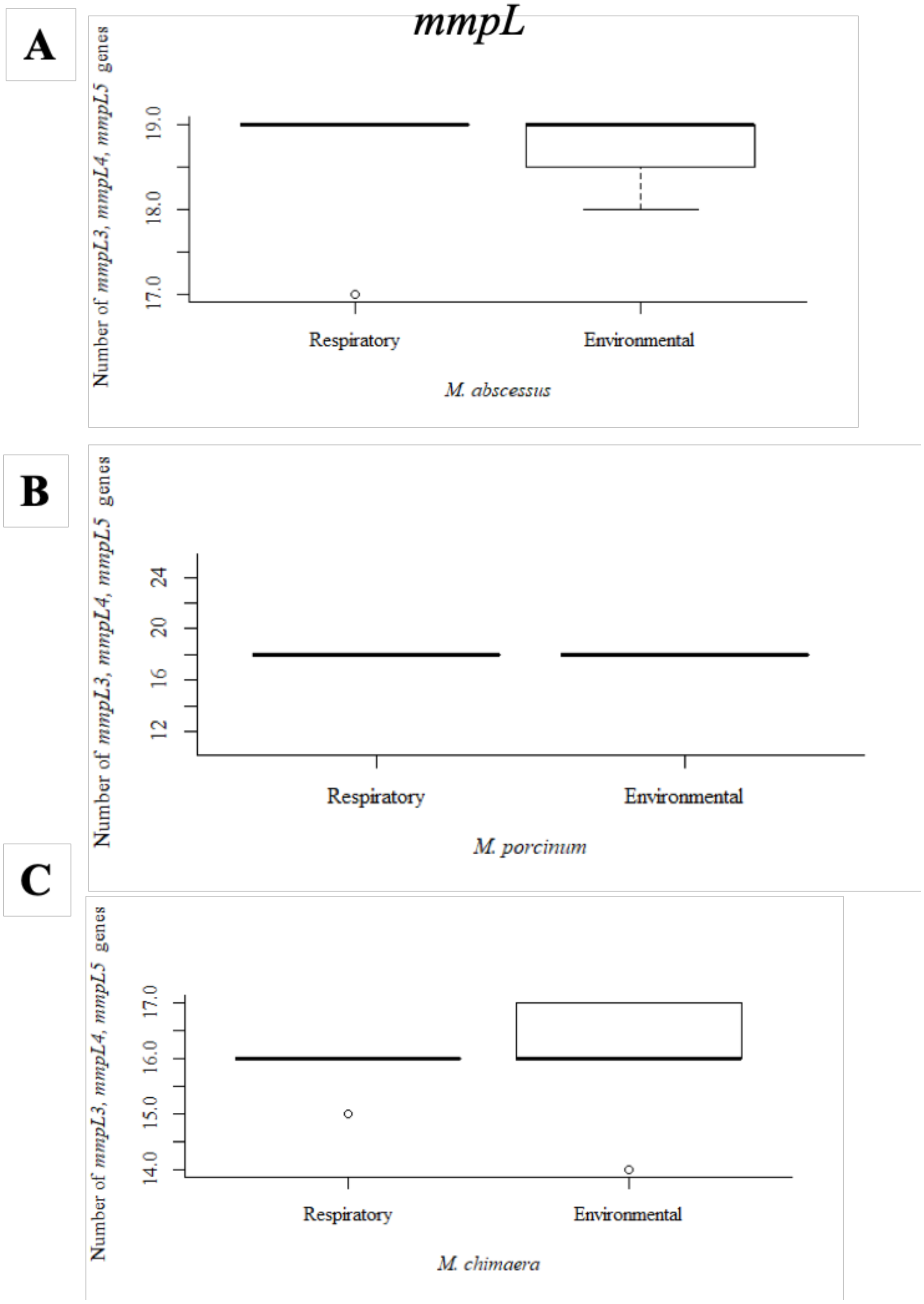

Supplementary Figure 5. Box plot showing the number of *mmpL345* genes in respiratory and environmental **A)** *M. abscessus;* **B)** *M. porcinum;* and **C)** *M. chimaera*.

	**# of times there was agreement between low iron and CAS results:**	**# of times there was disagreement between low iron and CAS results:**
All RGM (*i.e., M. abscessus* and *M. porcinum*)	22/28 (79%)	6/28 (21%)
All SGM (*i.e., M. chimaera*)	16/23 (70%)	7/23 (30%)

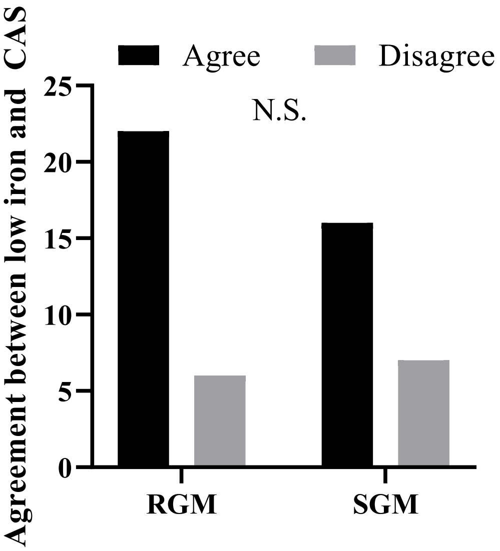

Supplementary Figure 6. Summarized outcomes between all NTM isolate groups tested in this study for outcome agreement between low iron and CAS assays. Top: Summary table based on outcomes shown in Supplementary Tables 3 and 4. Bottom: Data in top table was graphed. N.S. = not significant; $p = 0.5294$.

	**Agreement between low iron and CAS:**	**Disagreement between low iron and CAS:**
Respiratory RGM (*i.e., M. abscessus* and *M. porcinum*)	13/14 (93%)	1/14 (17%)
Environmental RGM (*i.e., M. abscessus* and *M. porcinum*)	9/14 (64%)	5/14 (36%)
Respiratory SGM (*i.e., M. chimaera*)	8/14, (57%)	6/14 (43%)
Environmental SGM (*i.e., M. chimaera*)	8/9 (89%)	1/9 (11%)

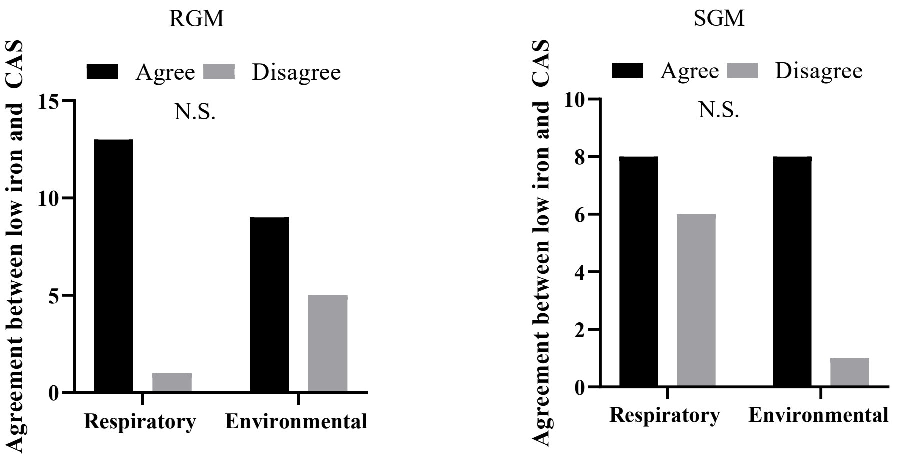

N.S., p = 0.1647

N.S., p = 0.1760

Supplementary Figure 7. Summarized results between all RGM and SGM isolates tested stratified by respiratory and environmental groups for outcome agreement between low iron results and CAS assays. Top: Summary table based on outcomes shown in Supplementary Tables 3 and 4. Bottom: Data in top table were graphed by RGM and SGM outcomes. N.S. = not significant; (RGM) p = 0.1647; (SGM) p = 0.1760.
